# “The mothers have eaten unripe grapes and the children's teeth are set on edge”: the potential inter-generational effects of the Holocaust on chronic morbidity in Holocaust survivors’ offspring

**DOI:** 10.1186/2045-4015-3-11

**Published:** 2014-03-25

**Authors:** Lital Keinan-Boker

**Affiliations:** 1 School of Public Health, Faculty of Social Welfare and Health Sciences, University of Haifa, Haifa, Israel; 2 Israel Center for Disease Control, Ministry of Health, Gertner Institute, Sheba Medical Center, Ramat Gan, Israel

**Keywords:** Cohort, FOAD, Holocaust, Israel, National

## Abstract

Modern epidemiology has evolved in the last decades from the simplified “cause-effect” paradigm to a multi-factorial framework of causality. The concept of “Fetal Origin of Adult Diseases” (FOAD) is a good example: it suggests that preconception circumstances and fetal exposures as well as infancy and early childhood experiences may eventually change an individual’s susceptibility to adult morbidity through fetal programming and epigenetic changes. The FOAD concept was supported, between others, by well-designed cohort studies carried out on non-Jewish World War II (WWII) survivors, exposed to hunger during the War years. However, data on late physical morbidity of Jewish WWII survivors are still scarce.

The current paper presents some cohorts addressing the FOAD hypothesis in relation to the long-term impact of early exposures to hunger and their main results. It stresses the need for the establishing of a similar cohort in Israel, in order to study the long-term effects of the Holocaust on the health of Holocaust child survivors and on that of the “second” and “third” generations. A framework for such a cohort in Israel is also proposed.

Establishing a cohort of this character in Israel should be a national priority and policy. First, taking special care of Holocaust survivors is a somewhat neglected national obligation. Second, if the population of Holocaust survivors and their offspring is indeed a high risk group for late chronic morbidity, higher awareness may lead to better primary prevention and to tailored secondary prevention programs. Third, the population at stack is unique and its contribution to the consolidation of the FOAD theory and its translational applications may be of foremost importance, in the global and national sense.

## Background

Modern epidemiology has evolved through several eras so far. The “Sanitary statistics” era (first half of the 19^th^ century) was followed by the “Infectious disease” era (late 19^th^ century through first half of 20^th^ century) and the “Chronic disease” era (second half of the 20^th^ century). The last of these focused primarily on immediate risk factors and was often characterized by the “black box paradigm” that is relating exposures to outcomes but not always taking into account intervening factors or patterns of pathogenesis. However, the complexities of chronic disease research dictated a need to move from the specific cause model anchored in the “Infectious disease” era, to new, multi-causal concepts of etiology [1].

The “Thrifty gene theory” introduced in 1962 by James V. Neel [2], offered a gene-environment interaction as an explanation for the growing incidence of diabetes mellitus. According to this theory, genes which predispose to diabetes (‘thrifty genes’) were historically advantageous since they enabled individuals to collect and process food efficiently to deposit fat during periods when food was abundant in preparation for periods of food scarcity. In modern world, however, where food is constantly available, these same genes had a detrimental effect, paving the way to increased chronic morbidity. The theory was soon expanded to encompass obesity, and even though criticized and flawed, it had the significant advantage of addressing potential intervening factors in the exposure-outcome paradigm rather than focusing solely on risk factors [2].

The new theoretical framework of Fetal Origin of Adult Diseases (FOAD) published by David JP Barker [3] in 1992, focused on prenatal growth patterns reflected in low birth weight, and suggested that fetal experiences may determine the long-term risk for a variety of chronic diseases (coronary artery disease, hypertension, obesity and insulin resistance), through fetal programming [3]. Poor nutritional conditions for the pregnant mother cause biologic and metabolic adaptations of the fetus which serve to prepare it for survival in an environment in which resources are scarce. The term “Thrifty phenotype”, coined in response to the “Thrifty gene” theory, implies that these adaptations are not genetic but metabolic and physiological [4]. Recently, the “Thrifty Epigenomic Hypothesis”, a combination of the Thrifty Phenotype and Thrifty Genotype hypotheses, was proposed [5]. While this hypothesis accepts that there is genetic predisposition for being “thrifty”, it argues that an individual’s risk for disease is primarily determined by subtle, epigenetic modifications at many genomic loci in response to prenatal and environmental influences, inducing higher susceptibility for complex morbidity such as the Metabolic Syndrome in the long run [5].

The FOAD theory is based on the biological fact that one genotype may give rise to different phenotypes, depending on environmental and other influences during development. The benefit of this phenotypic plasticity is that under changing environmental conditions, it ensures the development of a phenotype which is best matched to the current environment [6]. Thus, when malnutrition occurs during fetal development as reflected in low birth weight, it may be compensated for by accelerated growth thereafter. The exposure to malnutrition during fetal life and the rapid childhood growth pattern that follows are apparently accompanied by specific metabolic and physiological responses which alter the susceptibility to cardiovascular diseases and type II diabetes mellitus in adult life [6,7]. In animals, rapid/compensatory growth responses include shortening of the protective ends of the chromosomes (the telomeres) and increased cell death and organ degradation, which offer a potential explanation for certain associations between low birth weight and morbidity observed in humans [6]. The association may be explained by several mechanisms; first, lower birth weight may indicate less functional capacity in key organs, such as the kidney, which may later lead to hypertension [7]. Second, the fetal/infancy/childhood exposure may influence the setting of certain hormones and metabolic pathways (“thrifty phenotype”), assuring appropriate blood glucose concentration in the brain at the expense of other organs (i.e., muscles) [7]. Third, people who were small at birth may be vulnerable to chronic morbidity later in life due to non-biological influences, such as low socioeconomic status [7]. Oxidative stress programming may be another potential explanation. Genetic susceptibility, environmental and nutritional factors, gestational diabetes and hypertension may cause elevated oxidative stress that if experienced in critical time windows during early development may affect, directly or indirectly, growth patterns and gene expression, and eventually also adult susceptibility for chronic conditions. Some experimental evidence supports the oxidative stress theory and if approved, it may be tested by means of intervention trials with antioxidant supplementation [8].

Although criticized from some quarters, the FOAD hypothesis has expanded to include not only conditions associated with the Metabolic Syndrome but also with nervous system diseases, mental health and cognitive function [9]. Its main strength is its ability to conceptualize the linkage between genetic, environmental and individual determinants in different life stages to predict adult morbidity. In this respect, it is in agreement with Rothman’s model of causality, which suggests that different contributing determinants (i.e., genetic, environmental, social and others) must accrue throughout the lifespan in order to create a “sufficient cause” for the development of a disease [10].

Some of the data that support the FOAD hypothesis were derived from studies addressing outcomes of war-related famine exposures, such as those occurring during the Second World War (WWII) in Europe, thus the current literature was screened to trace relevant cohorts.

### The Dutch studies

The Dutch famine, lasting from October 1944 through May 1945, was the result of the blockading of shipment of food supplies to the occupied Dutch territories by the Germans in retaliation for a railway strike. The famine had been further exacerbated by the lack of means of transportation and by an extremely cold winter. At the peak of the famine, daily caloric intake dropped to as low as 500-600 Kcal per person. The famine ended abruptly upon the surrender of the German forces in the Netherlands on May 5^th^, 1945 [11].

The first study addressing prenatal exposure to the Dutch famine and adult health outcomes was carried out in the 1970’s and employed a cohort of 400,000 18 year old male army recruits, born before, during or after the “hunger winter”. Later studies used cohorts of children born during the famine or immediately after; in some of these studies the controls used were same-sex siblings, i.e., non-exposed brothers and sisters of exposed individuals. Other studies were based on volunteers from the general population; ‘exposure’ was defined primarily using ecological determinants, e.g., place and time of birth [11].

The results of the various studies indicated that prenatal exposure to famine was associated with an average drop of about 300 grams in birth weight, in higher rates of adult obesity, impaired glucose metabolism and schizophrenia, implying the existence of intergenerational effects [11]. Results regarding hypertension, hyperlipidemia and coronary artery disease are regarded as controversial [11].

The main strength of these studies is the well-defined period of exposure and the detailed information available on nutritional intake based on national ration data. However, the main weakness in some of these studies is the ecological basis (time of birth; place of birth) used for the exposure status definition.

### The siege of Leningrad

The Siege of the city of Leningrad by the German army began on September 8^th^, 1941, and lasted 872 days, until January 27^th^, 1944. The purpose of the Siege was not only to conquer the city, but also to break the spirit of its population and to decimate it. In addition to preventing food supplies from reaching Leningrad, the German troops steadily bombarded it from the ground and from the air. The most severe food shortage took place on winter 1941–42, when official rations for ‘dependents’ dropped to 460 Kcal/person/day but in reality were closer to 300 Kcal/day, and contained virtually no protein. Food shortages eased somewhat following that winter, thanks to a smuggling route across Lake Ladoga, but severe malnutrition persisted. It is estimated that out of the 2.7 million individuals who lived in Leningrad at the beginning of the Siege, 0.7-0.8 million died of hunger and war traumas [12].

Early studies that looked at the short-term consequences of the Siege of Leningrad on adults, revealed a hypertension epidemic, which has been attributed to the post-starvation compensatory feeding syndrome. In addition, a steep drop in birth weight - by an average of 500-600 gr - was reported [12].

A 1973 “détente”-initiated US-Soviet agreement, the Lipid Research Clinics Collaboration, paved the way for Shestov (himself a survivor of the Leningrad Siege) and colleagues to study the mid-range and long-term consequences of this exposure to famine. Between 1975 and 1982 the researchers formed a cohort comprised of inhabitants of Leningrad (now St. Petersburg) whose exposure status was based on whether or not they reported being in the city during the siege. In total, the ‘exposed’ group included 3,900 men born between 1916–1935 and 1,429 women born between 1910–1940. This cohort was followed through 2005 and is currently referred to as “the St. Petersburg Cohort”. In addition, Stanner et al. carried out a cross-sectional study on 169 survivors who were exposed *in utero*, 192 survivors who were exposed in infancy, and 182 non-exposed controls [12].

The results of the small cross-sectional study on *in-utero* and infancy exposure indicated a significantly higher diastolic blood pressure in the exposed vs. non-exposed subjects, but the differences between these groups on other measures (e.g., glucose and lipid metabolism, diabetes, circulatory disease) were non-significant [12]. The main weaknesses of this study are its cross-sectional design which does not allow causal inference and the small sample size which limits its power.

The results of the St. Petersburg Cohort, which focused on the differential effects of exposure at varying ages, starting from age 6 in men and age 1 in women, showed that exposure, particularly during puberty, was positively associated with hypertension, ischemic heart disease and mortality due to breast cancer in women. The clear effect of age at exposure on the outcomes suggests that re-programming of physiological systems occur at specific age windows and may be modified by gender. The researchers concluded that further research is needed in order to explore the potential inter-generational outcomes of exposure to famine [12]. The main strength of this cohort is its large number of participants. Its weaknesses include a large potential for a selection bias (survival bias) due to the severity of the famine and its long duration, an exposure definition based on self-report and thus prone to misclassifications and the fact that subjects exposed prenatally or at early infancy (males) were not included.

### British channel islands

The British Channel islands of Jersey and Guernsey were invaded by the German army in summer 1940. About one quarter of the islands’ population left or was evacuated before the occupation, which lasted through May of 1945. Between August, 1944, and May 9^th^, 1945, when they were liberated, the islands had no access to external food supplies. The islanders’ limited food intake, averaging 1,500 Kcal/day/person during the occupation years, quickly dropped to around 1,100, and would have dropped further if not for the assistance provided by the International Committee of the Red Cross [13].

Building on the ‘Dutch Famine’ and the ‘Leningrad Siege’ studies and on the FOAD hypothesis, three separate birth cohorts of Channel Islanders were established, with the aim of examining health outcomes in later life due to exposure to the Occupation *in utero,* infancy, childhood, adolescence and young adulthood. These “Channel Islands Occupation Birth Cohorts” include (1) the “British Registration Cohort” established in 1987, which involved linking of Guernsey male birth registration data to screening health data recorded by the Guernsey Chest and Heart Association; (2) the “Volunteer Cohort” established in 2001, which used self-report data from volunteers in the Channel Islands to examine the relationship between life course events and later health status; and (3) the “Midwife cohort”, which linked pre-war (1923–1937) midwifery records from the Islands to other sources of health data. Returning evacuees served as an appropriate comparison group in some of the studies [13].

The results of the studies carried out so far are limited, due to small numbers of participants and potential self-selection effects. Nevertheless, strong evidence for an association between exposure at early life and cardiovascular disease and adult mortality, and some evidence on elevated risk of metabolic disorders and obesity, were obtained [13]. In spite of their limitations, the strength of the “Channel Islands Occupation Birth Cohorts” is their diverse information sources which allow for data linkage and validation. The research effort is continuing to investigate a variety of different study questions.

### The Chinese cohorts

In China, the economic and social policies instituted between 1958 and 1961, collectively known as the Great Leap Forward, aimed at the rapid transformation of the Agrarian society of the time into an industrialized, communist one. These policies, combined with a sudden drop (by 15%) in the national grain output in 1959, resulted in the worst famine experienced in history. The Great Famine of China lasted from 1959 through 1961 and killed between 16 and 30 million people [14]. For the purpose of comparison, it is noted that the death toll of WWII is estimated to have reached a total of 80 million.

Since 1987, over 17 historical cohorts were established for the purpose of studying the long-term impact of exposure to the Chinese famine. The cohorts varied in sample size (from hundreds to over half a million participants), in methodology and in the outcomes studied (height, overweight and obesity, glucose metabolism and diabetes, hypertension, metabolic syndrome, quality of life, schizophrenia, socioeconomic parameters etc.). Most studies, if not all, assessed exposure to famine by means of proxy variables (i.e., place of residence, year of birth). Consistent associations were observed between fetal exposure to famine and diabetes, hypertension and the Metabolic Syndrome in adult life, as well as obesity in women [14]. The potential for a selection bias, taking into account the severity of the exposure, is common to all cohorts. The fact that these cohorts only now reach the age where chronic morbidity is more prevalent may also hamper the ability to trace significant associations. However, the large numbers involved are a definite strong point. The research continues today.

## Case for action

Holocaust survivors were exposed to severe famine and to extensive mental and physical stress for prolonged time periods and have experienced horrific hardships throughout WWII [15,16]. It has been suggested that these experiences may have increased the survivors’ vulnerability to mental and physical morbidity in later life [17], in particular, osteoporosis [18] and cancer [19]. A recent cross-sectional pilot survey on Holocaust survivors born in Occupied Europe in 1940–1945 found that they had higher prevalence of obesity, dyslipidemia, hypertension, diabetes and cardio-vascular diseases compared to their non-exposed counterparts [20]. It is not yet clear whether the children of Holocaust survivors, the “second generation”, are at a higher risk for certain chronic diseases due to an inter-generational impact, or whether, in other words, the Biblical proverb “the fathers (or in this case, the mothers) have eaten unripe grapes and the children’s teeth are set on edge” is valid in this case as well [21].

Based on the results of the cohort studies described earlier, the establishing of an Israeli national cohort to study the long-term health impact of the Holocaust on the survivors and their descendants, i.e., the “second” and perhaps also the “third” generations, is long overdue and should be incorporated into the policy of the Israeli Government and be highly prioritized, in light of the national commitment and the public sensitivity to matters concerning Holocaust survivors and their offspring. The extreme exposures associated with the Holocaust and the fact that currently the largest community of Holocaust survivors resides in Israel [22] combined with the time elapsing since then that allows for the study of the ‘second’ and perhaps also the ‘third’ generations, makes an Israeli study of this sort inimitable. Such an Israeli cohort is expected to yield novel and rich scientific data that may clarify controversial findings, not only because of the uniqueness of the study population and its exposure, but also because the cohort study is meant to include all eligible subjects, thus to minimize potential selection bias, and to be based on “hard data” with respect to health outcomes, which may decrease the potential for an information bias. Its results may serve as sound evidence for health policy guidelines. Indeed, unfortunately none of the cohorts described earlier lead to a specific health policy recommendation as yet, although they may have, especially the Dutch and the Leningrad cohorts, even if only to draw a high risk population profile and to suggest certain screening strategies. However, several other research topics, investigated through cohort and intervention studies, did introduce a substantial change in health policy and even legislation. For example, Modan’s cohort study disclosed an association between irradiation treatment for tinea capitis and head and neck tumors [23], which defined a high-risk group in the Israeli population, and in 1994 also lead to the legislation of the Law for the Compensation of Tinea Capitis Patients in Israel. Another, more global, example is the many cohorts and intervention studies on diet and cardiovascular diseases [24] which lead to the widely-adopted recommendation of the Mediterranean Diet by both professional organizations and policy-makers.

The establishment of such an Israeli cohort is of particular value to the Israeli Ministry of Health, given the potential impact its findings may have on the health system should a higher risk profile for chronic morbidity be identified. In this case, primary prevention as well as early detection and intervention options may be available and relevant recommendations for policy makers can be formulated. First, a global message, perhaps in the form of a health campaign, may address all relevant subjects, presenting the findings and stressing the importance of primary prevention with regard to most chronic diseases (i.e., smoking cessation, healthy nutrition, active lifestyle etc.). Second, secondary prevention may be practiced by the identification of high-risk subjects either through an algorithm that takes into account their date of birth, place of birth, parents’ place of birth and date of immigration, operated by the computerized databases of the Health Maintenance Organizations (HMOs) or through direct questions by the primary care providers. Once identified, these subjects may be actively reached out and encouraged to perform the common age- and gender-matched screening tests [25] which are not always performed as recommended, in an attempt to assess the subjects’ risk potential for certain diseases and to enable their early detection. The implementation of these recommendations may be monitored through the National Program for Quality Indicators in Community Healthcare in Israel, which is an initiative of the Israeli Ministry of Health and the four Israeli HMOs, aimed to evaluate the quality of community-based medical care in Israel, including improvements and modifications to the healthcare system introduced over time, and variations in quality of care between subgroups [26]. Thus, such a research cohort followed by practical recommendations and implementation may eventually assist in lowering the burden of non-communicable diseases and their treatment in Israel.

## Proposal

The Helsinki cohort, established by Barker and Eriksson [27], may provide a good model for a national Israeli cohort. It comprises men (n = 3,639) and women (n = 3,447) who were born at the Helsinki University Central Hospital between 1924 and 1933, went to school in Helsinki and were still living in Finland in 1971, when a personal identification number (PIN) was allocated to each Finnish citizen. Detailed birth and school health records were available for all participants [27]. The PIN enabled the linking of different national registries (i.e., those recording hospitalizations, treatment for chronic medical conditions, incidence of diabetes) yielding data on chronic morbidity later in life. The cohort was later expanded to include men and women born at the Helsinki University Central Hospital between 1934 and 1944, who were still living in Finland on 1971 (a total of 4,630 men and 4,130 women). Birth records, child welfare clinic records (including repeated measurements of height and weight) and school health records were abstracted and the data were linked with national registries to trace the incidence of chronic morbidity [28,29]. This cohort also made possible the study of a unique population: between 1939 and 1945 Finland fought three wars: the Winter War against the Soviet Union, the Continuation War with Germany against the Soviet Union, and the Lapland War against Germany. During this period, approximately 70,000 Finnish children were evacuated to temporary foster homes in Sweden and Denmark, unaccompanied by their parents. In order to facilitate the adjustment to their new homes and the assimilation into the new communities, siblings were usually separated. The evacuations were voluntary and were perceived as a positive opportunity by families across all socioeconomic classes. The Helsinki Birth Cohort allowed the study of the long-term effects of the evacuation on the psychological and physiological well-being of these children decades later [30].

An Israeli cohort of Holocaust survivors offspring (“second generation”) and a matching control group can be identified by using proxy variables such as place of birth, year of birth, parents’ origin and year of immigration, and can be traced by the Population Registry Database. Indeed, this definition of exposure is basically ecological, but it will encompass all eligible subjects in the Israeli population and thus misclassifications are expected to be non-differential. Once the cohort is identified, data on relevant health outcomes can be mined by linking the unique PIN allocated to all Israeli citizens since the establishment of the State of Israel in 1948, with information in the databases of the Israeli Health Maintenance Organizations, hospital discharge files and national registries (e.g., Israel National Cancer Registry, the End-Stage Renal Disease National Registry and the National Diabetes Registry) as well as the National Mortality Registry database. These linkages can be carried out periodically, adding information to the dataset created. This approach will minimize selection bias due to “loss to follow up” as well as information bias, since morbidity data will mostly be based on “hard data”. In addition, questionnaires may be sent to the participants to enrich the personal information. A subset of the cohort may be asked to consent to be interviewed, examined and measured, as well as to donate blood samples and buccal swabs for biomarker levels and DNA testing to identify epigenetic changes.

The initial expenses associated with establishing such a cohort may be covered by a research fund. However, maintenance of the cohort database should be a national priority and involve a basic annual budget dedicated only for that. Any further research questions aiming to make use of the initial data and expand it should be self-budgeted by research grants or other resources.

Studies in laboratory animals were able to show that epigenetic changes induced by stress to a pre-pregnant or pregnant mother, were carried forward not only to the immediate next generation, but also to those following it [31-33]. One of the explanations offered is that epigenetic changes affecting the embryo’s gametes may be transferred further also to the third generation. The cohort proposed may also be used to identify the “Third generation” and to further enable the study of an inter-generational impact of the Holocaust.

## Conclusions

Israel presents a unique research opportunity with respect to the establishing of a cohort of Holocaust survivors’ offspring, because of its small size, the rich data on health outcomes and the ability to trace these outcomes through linkage of databases via the use of PINs.

Such a cohort should be a national priority because of the uniqueness of the population in question, i.e., Holocaust survivors and their offspring whose health outcomes have barely been studied to date, perhaps out of a neglected national obligation. In addition, if the population of Holocaust survivors and their offspring is indeed a high risk group for late chronic morbidity, a policy of higher awareness may lead to better primary prevention and to tailored secondary prevention programs. Last but not least, as mentioned before, the population at stack is unique and its contribution to the consolidation of the FOAD theory and its translational applications may be of foremost importance, in the global and national sense.

As Calkins & Devaskar [34] stated, “…Implications of the FOAD [theory] extend beyond the low birth weight population and include babies exposed to stress, both nutritional and nonnutritional, during different critical periods of development, which ultimately result in a disease state. By understanding FOAD, health care professionals and policy makers will make this issue a high health care priority and implement preventive measures and treatment for those at higher risk for chronic diseases” [34]. This statement is especially true for the offspring of Holocaust survivors who reside in Israel.

## Competing interests

The author declares that she has no competing interests.

## Acknowledgements

The author would like to acknowledge the valuable contribution of Dr. Rena Mei-Tal to the linguistic formatting and editing of the manuscript.

## Authors’ information

The author (LKB) is a physician specializing in public health and a senior lecturer in the School of Public Health in the University of Haifa. Her main research interest is cancer epidemiology. She was involved with one of the Dutch Hunger Winter studies while studying for her PhD degree in the Netherlands. In Israel, she studied, together with other colleagues, the incidence of cancer in Holocaust survivors, and, in particular, the association between WWII-related hunger, WWII-related post-traumatic stress disorder and breast cancer. Recently she conducted two studies on the prevalence of late morbidity in Holocaust survivors born during WWII.

## References

[B1] Susser M Susser E Choosing a future for epidemiology: I. Eras and paradigms Am J Public Health 1996 86 668 673 10.2105/AJPH.86.5.668 8629717 PMC1380474

[B2] Neel JV Diabeteb mellitus: a thrifty genotype rendered “detrimental” by progress? Am J Hum Genet 1962 14 4 353 362 13937884 PMC1932342

[B3] Barker DJP Fetal and infant origin of adult disease 1992 London, UK: Br Med J ISBN 0-7279-0743-3

[B4] Hales CN Barker DJ Type 2 (Non-insulin dependent) diabetes mellitus: the thrifty phenotype hypothesis Diabetologia 1992 35 7 595 601 10.1007/BF00400248 1644236

[B5] Støger R The thrifty epigenotype: an acquired and heritable predisposition for obesity and diabetes? Bioassays 2008 30 2 156 166 10.1002/bies.20700 18197594

[B6] Barker DJP Eriksson JG Forsēn T Osmond C Fetal origin of adult disease: strength of effects and biological basis Int J Epidemiol 2002 31 1235 1239 10.1093/ije/31.6.1235 12540728

[B7] Barker DJP Osmond C Kajantie E Eriksson JG Growth and chronic disease: findings in the Helsinki Birth Cohort Ann Hum Biol 2009 36 5 445 458 10.1080/03014460902980295 19562567

[B8] Luo ZC Frazer WD Julien P Deal CL Audibert F Smith GN Xiong X Walker M Tracing the origins of “fetal origins” of adult diseases: programming by oxidative stress? Med Hypotheses 2006 66 38 44 10.1016/j.mehy.2005.08.020 16198060

[B9] Skogen JC Øverland S The fetal origins of adult disease: a narrative review of the epidemiological literature J R Soc Med Short Rep 2012 3 1 7 10.1258/shorts.2011.011079 PMC3434434 23301147

[B10] Rothman K Greenland S Causation and causal inference in epidemiology studies Modern Epidemiology 1998 Hauppauge, New York, US: Lippincot 7 28 chapter 8

[B11] Lumey LH van Poppel FWA Lumney LH, Vaiserman A The Dutch Famine of 1944–45 as a human laboratory: changes in the early life environment and adult health Early Life Nutrition and Adult Health and Development 2013 Hauppauge, New York, US: Nova Publishers Inc 59 76 Chapter 3

[B12] Vågerö D Koupil I Parfenova N Sparen P Lumney LH, Vaiserman A Long term health consequences following the Siege of Leningrad Early Life Nutrition and Adult Health and Development 2013 Hauppauge, New York, US: Nova Publishers Inc 207 225 Chapter 10

[B13] Ellison GTH Lumney LH, Vaiserman A The health in later life of channel islanders exposed to the 1940–45 occupation and Siege Early Life Nutrition and Adult Health and Development 2013 Hauppauge, New York, US: Nova Publishers Inc 77 108 Chapter 4

[B14] Li Y Hu FB Ma G Lumney LH, Vaiserman A Early life nutrition and adult health and development In Early life nutrition and adult health and development 2013 Hauppauge, New York, US: Nova Publishers Inc 133 154 Chapter 6

[B15] Shasha SM Morbidity in the ghettos during the Holocaust Harefuah 2002 141 4 308 364 408, 409 [Hebrew] 12017893

[B16] Shasha SM Morbidity in the concentration camps of the Third Reich Harefuah 2004 143 4 272 276 318 [Hebrew] 15116584

[B17] Shasha SM Livshitz A Ohry A Late morbidity among Holocaust survivors Harefuah 2009 148 4 224 228 278 [Hebrew] 19630342

[B18] Marcus EL Menczel J Higher prevalence of osteoporosis among female Holocaust survivors Osteoporos Int 2007 18 11 1501 1506 10.1007/s00198-007-0389-x 17492392

[B19] Keinan-Boker L Vin-Raviv N Liphshitz I Linn S Barchana M Cancer incidence in Israeli Jewish survivors of World War II J Natl Cancer Inst 2009 101 21 1489 1500 10.1093/jnci/djp327 19861305

[B20] Bercovich E Keinan-Boker L Shasha SM Long – term health effects in adults born during the holocaust Isr Med Assoc Jin press 24834754

[B21] Hazani E Shasha SM Effects of the Holocaust on the physical health of the offspring of survivors Isr Med Assoc J 2008 10 251 255 18548975

[B22] DellaPergola S Review of Relevant Demographic Information on World Jewry International Commission on Holocaust Era Insurance Claims 2003 Jerusalem, Israel at: http://www.claimscon.org/forms/allocations/Review_Della%20Pergola%20ICHEIC_.pdf (last accessed: March 2014)

[B23] Modan B Baidatz D Mart H Steinitz R Levin SG Radiation-induced head and neck tumours Lancet 1974 1 7852 277 279 4130470 10.1016/s0140-6736(74)92592-6

[B24] Serra-Majem L Roman B Estruch R Scientific evidence of interventions using the Mediterranean diet: a systemic review Nutr Rev 2006 64 2Pt2 S27 S47 16532897 10.1111/j.1753-4887.2006.tb00232.x

[B25] Tabenkin H Lahad A Clinical guidelines: the recommendations of the Israeli task force for health promotion and preventive medicine 2013 Israel Medical Association and the Association of Family Physicians in Israel. Israel [Hebrew]

[B26] Manor O Shmueli A Ben Yehuda A Paltiel O Calderon R Jaffe DH National program for quality indicators in community healthcare in Israel: report 2008–2010 The Ministry of Health, State of Israel; The Israel National Institute for Health Policy Research; The Health Council. June 2012, Israel. Website: http://www.israelhpr.org.il/ (last visited: Oct 2013)

[B27] Forsēn T Eriksson J Tuomihleto J Reunanaen A Osmond C Barker D The fetal and childhood growth of persons who develop type 2 diabetes Ann Intern Med 2000 133 3 176 182 10906831 10.7326/0003-4819-133-3-200008010-00008

[B28] Eriksson JG Forsēn TJ Tuomihleto J Osmond C Barker DJP Early growth and coronary heart disease in later life: longitudinal study BMJ 2001 322 949 953 10.1136/bmj.322.7292.949 11312225 PMC31033

[B29] Barker DJP Osmond C Forsēn TJ Kajantie E Eriksson JG Trajectories of growth among children who have coronary events as adults NEJM 2005 353 1802 1809 10.1056/NEJMoa044160 16251536

[B30] Alastalo H Räikkönen K Pesonen AK Osmond C Barker DJ Kajantie E Heinonen K Forsēn TJ Eriksson JG Cardiovascular health of Finnish war evacuees 60 years later Ann Med 2009 41 1 66 72 10.1080/07853890802301983 18720095

[B31] Shachar-Dadon A Schulkin J Lshem M Adversity before conception will affect adult progeny in rats Dev Psychol 2009 45 1 9 16 19209986 10.1037/a0014030

[B32] Franklin TB Russig H Weiss IC Gräff J Linder N Michalon A Vizi S Mansuy IM Epigenetic transmission of the impact of early stress across generations Biol Psychiatry 2010 68 408 415 10.1016/j.biopsych.2010.05.036 20673872

[B33] Stewart RJ Preece RF Shepard HG Twelve generations of marginal protein deficiency Br J Nutr 1975 33 233 253 10.1079/BJN19750027 1115762

[B34] Calkins K Devaskar SU Current problems in pediatric and adolescent Health Care 2011 41 6 158 176 10.1016/j.cppeds.2011.01.001 PMC4608552 21684471

